# Occurrence of Soil Fungi in Antarctic Pristine Environments

**DOI:** 10.3389/fbioe.2019.00028

**Published:** 2019-03-07

**Authors:** Paola Durán, Patricio J. Barra, Milko A. Jorquera, Sharon Viscardi, Camila Fernandez, Cristian Paz, María de la Luz Mora, Roland Bol

**Affiliations:** ^1^Scientific and Technological Bioresource Nucleus, Universidad de La Frontera, Temuco, Chile; ^2^Biocontrol Research Laboratory, Universidad de La Frontera, Temuco, Chile; ^3^Laboratorio de Ecología Microbiana Aplicada, Departamento de Ciencias Químicas y Recursos Naturales, Universidad de la Frontera, Temuco, Chile; ^4^Departamento de Procesos Diagnósticos y Evaluación, Facultad de Ciencias de la Salud, Universidad Católica de Temuco, Temuco, Chile; ^5^Agrosphere (IBG-3), Institute of Bio- and Geosciences, Forschungszentrum Jülich, Jülich, Germany

**Keywords:** Antarctica, fungal community, biodiversity index, extreme environment, cold desert

## Abstract

The presence of fungi in pristine Antarctic soils is of particular interest because of the diversity of this microbial group. However, the extreme conditions that coexist in Antarctica produce a strong selective pressure that could lead to the evolution of novel mechanisms for stress tolerance by indigenous microorganisms. For this reason, in recent years, research on cold-adapted microorganisms has increased, driven by their potential value for applications in biotechnology. Cold-adapted fungi, in particular, have become important sources for the discovery of novel bioactive secondary metabolites and enzymes. In this study, we studied the fungal community structure of 12 soil samples from Antarctic sites, including King George Island (including Collins Glacier), Deception Island and Robert Island. Culturable fungi were isolated and described according to their morphological and phenotypical characteristics, and the richness index was compared with soil chemical properties to describe the fungal community and associated environmental parameters. We isolated 54 fungal strains belonging to the following 19 genera: *Penicillium, Pseudogymnoascus, Lambertella, Cadophora, Candida, Mortierella, Oxygenales, Geomyces, Vishniacozyma, Talaromyces, Rhizopus, Antarctomyces, Cosmospora, Tetracladium, Leptosphaeria, Lecanicillium, Thelebolus, Bjerkandera* and an uncultured Zygomycete. The isolated fungi were comprised of 70% Ascomycota, 10% Zygomycota, 10% Basidiomycota, 5% Deuteromycota and 5% Mucoromycota, highlighting that most strains were associated with similar genera grown in cold environments. Among the culturable strains, 55% were psychrotrophic and 45% were psychrophilic, and most were Ascomycetes occurring in their teleomorph forms. Soils from the Collins Glacier showed less species richness and greater species dominance compared with the rest of the sites, whereas samples 4, 7, and 10 (from Fildes Bay, Coppermine Peninsula and Arctowski Station, respectively) showed greater species richness and less species dominance. Species richness was related to the C/N ratio, whereas species dominance was inversely related to C and N content. Thus, the structure of the fungal community was mainly related to soil chemical parameters more than sample location and altitude.

## Introduction

Antarctica is considered the “Land of Peace and Science” because it is the most extreme environment on the planet and represents an interesting and unique habitat for the colonization and survival of natural life. For these reasons, Antarctica is considered an “outdoor laboratory” where we can study different life forms subjected to multiple extreme conditions. The prevalent extreme conditions in Antarctic are low temperature, lack of water availability (cold desert) and precipitation, numerous freeze–thaw cycles, strong wind levels and high sublimation, evaporation and ultraviolet radiation (Selbmann et al., 2007). For this reason, it is very likely that strong selective pressures may have led to the evolution of still unknown mechanisms for stress tolerance by indigenous microorganisms.

Similar to other environments, among the biota present in Antarctica, the microbial life is mainly represented by archaea, bacteria, and fungi (Teixeira et al., 2013; Purves et al., 2016). However, fungi are the most diverse group in the different Antarctic ecosystems, including the soils (Godinho et al., 2015). The survival of fungi in extreme environments is a consequence of both ecological selection and evolutionary adaptations expressed at physiologic, metabolic, structural and genetic levels (Cowan et al., 2014). Selbmann et al. (2007) stated that Antarctic fungi could be cosmopolitan, where some propagules could be transported externally but are unable to grow under Antarctic conditions, while other indigenous well-adapted fungi, mainly psychrotolerants, are able to grow and reproduce even at low temperatures. Both psychotrophic and psychrophilic fungi have the ability to grow at 0°C. Psychotrophic fungi have a maximum growth temperature above 20°C, whereas psychrophilic fungi have an optimum growth temperature of 15°C or lower and a maximum growth temperature of 20°C (Robinson, 2001).

Some studies have determined that these specialized microorganisms are able to tolerate a wide range of stresses, including desiccation, hypersalinity, solar radiation, and low temperatures, by developing functional strategies, such as the production of bioactive compounds (Godinho et al., 2013), cold-active enzymes and antifreeze proteins (Robinson, 2001; Krishnan et al., 2011, 2018). In fact, Pacelli et al. (2017) characterized the effects of spaceflight-relevant radiation on the cryptoendolithic black fungus *Cryomyces antarcticus*, noting that the fungus maintained high survival and metabolic activity with no detectable DNA and ultrastructural damage, even after the highest dose of radiation. To date, the *Cryomyces* genus is considered one of the best eukaryotic models for astrobiological studies and has been used as a model for space experiments over the last decade (Coleine et al., 2018). However, although fungi represent the main microbial group in Antarctica, they are rarely studied (Selbmann et al., 2007).

In this context, Ding et al. (2016) studied the diversity and biological activities of 150 cultivable fungal strains isolated from Fildes Bay (King George Islands), where 18 isolates produced biologically active compounds. In contrast, Siciliano et al. (2014) characterized fungal and bacterial communities from a broader range of soil conditions, highlighting the importance of edaphic factors in controlling microbial communities, especially fungal communities. Because the vast majority of studies about microbial communities have been confined to bacteria (Maida et al., 2015; Cid et al., 2016; Tedesco et al., 2016; Sannino et al., 2017), very little is currently known about the ecology of fungi inhabiting the Antarctic continent.

Therefore, in this study, we focus on determining, analyzing, and comparing the fungal community structure and composition of twelve Antarctic soils from King George Island (including the Collins Glacier), Deception Island and Robert Island. In addition, culturable fungi were isolated and described according their morphological and phenotypical characteristics.

## Materials and Methods

### Sampling

**Figure 1:**
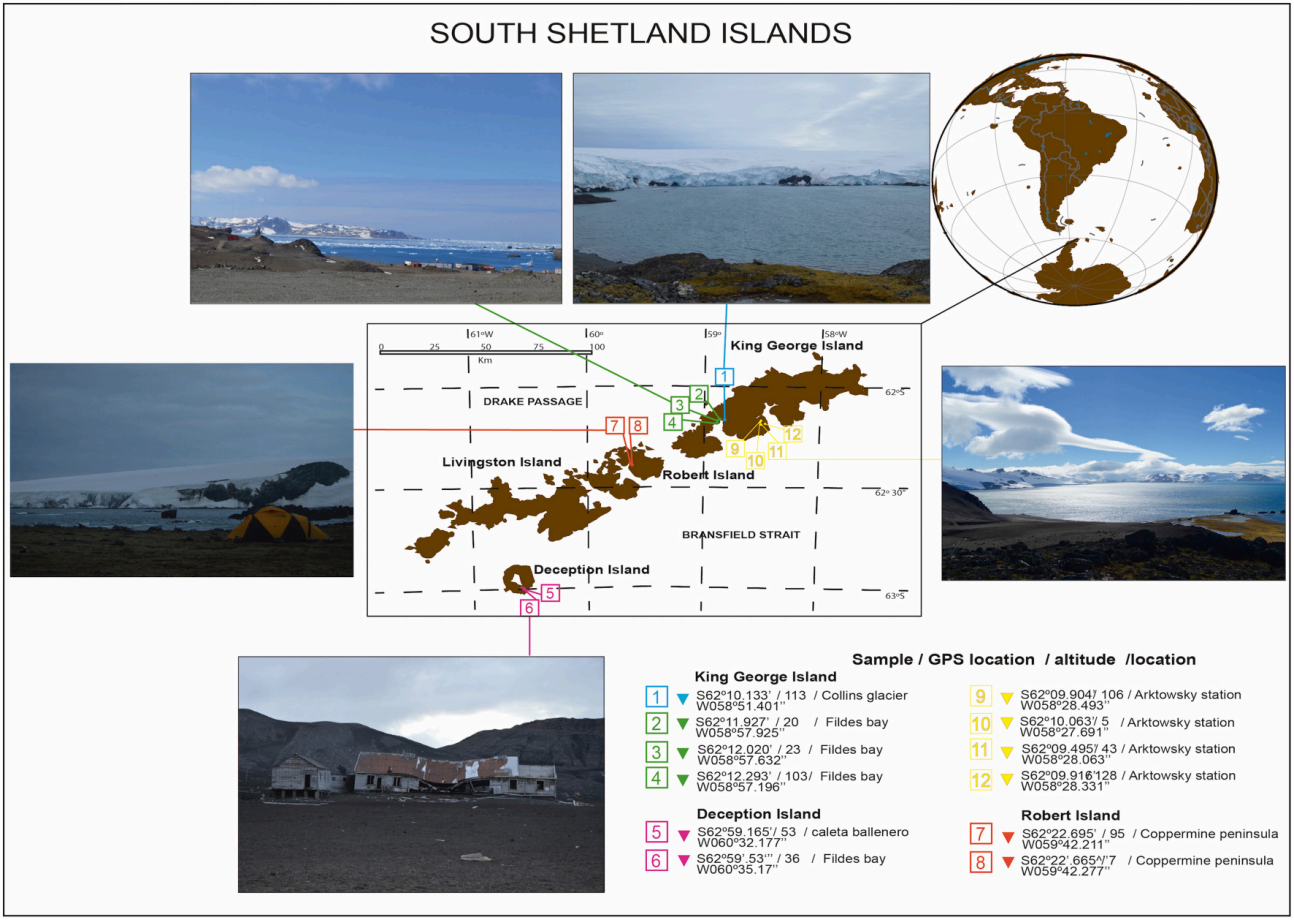
Antarctic soil sampling from the South Shetland Islands during expedition ECA53.

Bulk soil (without plant influence) samples were collected from South Shetland Island (62°00′S, 59°30′W) during the Antarctic campaign ECA-53, during the austral summer (2017) (Table 1). Twelve samples were collected from the top 0–20 cm without the presence of plants (bulk soil). Sample 1 corresponds to the Collins Glacier; samples 2, 3 and 4 correspond to Fildes Bay; samples 5 and 6 correspond to Deception Island; samples 7 and 8 correspond to Coppermine; and samples 9, 10, 11, and 12 correspond to Arctowski Station (Figure 1). The samples were transported in coolers (~4°C) and processed at the Laboratory of Research in Biocontrol located in Universidad de la Frontera.

**Table 1:** Chemical parameters of Antarctic soil samples.

**Soil**	**C (%)**	**N (%)**	**C/N**	**pH**
Soil 1	2.65 ± 0.29^a^	0.36 ± 0.001^a^	7.36 ± 0.81^c^	6.13 ± 0.30^g^
Soil 2	1.03 ± 0.04^b^	0.08 ± 0.000^d^	12.5 ± 0.48^bc^	6.53 ± 0.08^f^
Soil 3	0.27 ± 0.01^d^	0.02 ± 0.001^g^	15.8 ± 1.53^b^	6.73 ± 0.13^c^
Soil 4	0.46 ± 0.05^cd^	0.01 ± 0.002^h^	42.1 ± 4.46^a^	7.23 ± 0.22^a^
Soil 5	0.34 ± 0.04^d^	0.04 ± 0.001^e^	8.63 ± 1.05^bc^	6.74 ± 0.14^c^
Soil 6	0.21 ± 0.01^d^	0.04 ± 0.001^e^	5.92 ± 0.15^c^	5.88 ± 0.18^i^
Soil 7	1.13 ± 0.05^b^	0.20 ± 0.001^b^	6.23 ± 0.30^c^	5.46 ± 0.23^k^
Soil 8	0.25 ± 0.01^d^	0.04 ± 0.001^e^	6.96 ± 0.33^c^	5.61 ± 0.25^j^
Soil 9	0.79 ± 0.01^bc^	0.11 ± 0.001^c^	7.42 ± 0.18^c^	5.98 ± 0.02^h^
Soil 10	0.10 ± 0.00^d^	0.01 ± 0.001^h^	7.47 ± 0.44^c^	6.58 ± 0.15^e^
Soil 11	0.14 ± 0.01^d^	0.03 ± 0.001^f^	5.53 ± 0.64^c^	6.62 ± 0.04^d^
Soil 12	0.26 ± 0.01^d^	0.04 ± 0.000^e^	7.21 ± 0.62^c^	6.77 ± 0.13^b^

*Tukey test to compare different soil samples, values followed by the same letter do not differ at P < 0.05 (n = 3)*.

The chemical properties of the soil samples were determined as follows: carbon and nitrogen concentrations were determined by combustion in a Vario MicroCube elemental analyzer (DIN ISO 10694; Elementar Analysen systeme, Hanau, Germany) according to DIN ISO 10694 (1996). Samples were air dried and finely ground with a mortar and pestle before measurement. Samples did not contain inorganic C; hence, TC equals OC. Soil pH was measured in 1:2.5 soil/deionized water suspensions.

### Isolation of Culturable Fungi

Fungi were isolated from each soil sample according to Gonc et al. (2015). Briefly, 1 g of each sample was added to 9 mL of sterile saline solution (0.85% NaCl) in triplicate and vortexed. One hundred microliters of homogenized soil dilutions at 10^0^ and 10^−2^ were spread onto YM media (0.3% yeast extract, 0.3% malt extract, 0.5% peptone, 2% glucose, 2% agar, pH 6.2 ± 2). Plates were supplemented with chloramphenicol (100 μg mL^−1^) to prevent bacterial growth. The plates were incubated at 4, 15, and 25°C for 30 days to evaluate the optimal growth temperature of each fungal strain to determinate the existence of psychrophilic and/or psychrotrophic fungi. Pure cultures were visualized by scanning electron microscopy (VP-SEM) with an energy dispersive X-ray spectrometer detector (EDX, Hitachi, Japan).

### Identification of Fungal Strains

Genotypic characterization of selected fungal strains was performed based on sequencing of the ribosomal internal transcribed spacer 2 (ITS2) region. ITS2 was amplified by touchdown polymerase chain reaction (PCR) with the primer set fITS9 (5′-GAACGCAGCRAAIIGYG-3′) and ITS4 (5-′TCCTCCGCTTATTGATATGC-3′) as described by Ihrmark et al. (2016), using the following conditions: an initial denaturation at 95°C for 3 min; followed by 25 cycles each at 95°C for 30 sec; an annealing step with a 0.5°C decrease each cycle from 65 to 52.5°C; and extension at 72°C for 30 sec. Twenty-five additional cycles were carried out with denaturation at 95°C for 30 sec; a 55 °C annealing step; and primer extension at 72°C for 30 sec; with a final extension step of 7 min at 72°C. The PCR products were purified and sequenced by Austral-Omics (Universidad Austral of Valdivia-Chile). The sequence was compared with those present in the GenBank database and were deposited in the GenBank nucleotide sequence data library under the accession numbers in Table 2.

**Table 2:** Fungal strains isolated from South Shetland Island.

**Strain**	**Closest relatives or cloned sequences (accession N°)**	**Similarity (%)**	**Accesion N°**
*Penicillium* sp.SA1.3	*Penicillium commune*, wine cellar fungi (KT316690)	91	MG754011
*Pseudogymnoascus* sp.SA1.4	*Pseudogymnoascus pannorum*, cave bear bones (KY465766)	97	MG754012
*Penicillium* sp.SA2.2	*Penicillium brevicompactum*, cold environments of Western Himalaya (AM948959)	96	MG845129
*Lambertella* sp.SA3.1	*Lambertella viburni*, molecular phylogenetic studies (AB926098)	98	MG845130
*Cadophora* sp.SA3.2	*Cadophora malorum*, King George Island, Antarctic (MG813381)	99	MG754013
*Cadophora* sp.SA3.3	*Cadophora* sp. King George Island, Antarctic (MG813382)	99	MG754014
*Candida* sp.SA3.5	*Candida zeylanoides*, Pomegranate Fruits (KY366245)	95	MG754015
Uncultured Zygomycete SA3.6	*Fungal* sp, Antarctic Peninsula (FJ236010)	100	MG845131
*Mortierella* sp.SA3.7	*Mortierellaceae* sp, Antarctic Peninsula (HM589297)	100	MG754016
*Cadophora* sp.SA3.8	*Cadophora* sp., roots of *Populus deltoids* (KF428355)	88	MG754017
*Mortierella* sp.SA4.1	*Mortierella* sp., Antarctic Peninsula (MG00140)	88	MG754018
*Onygenales* sp.SA4.4	*Onygenales* sp., altitude lakes in the Indian trans-Himalayas (MF326613)	99	MG754019
*Geomyces* sp.SA4.5	*Geomyces* sp. King George Island, Antarctic (MG813416)	96	MG754020
*Vishniacozyma* sp.SA4.6	*Vishniacozyma victoriae*, Antarctic Peninsula (LC203739)	99	MG754021
*Talaromyce*s sp.SA4.7	*Talaromyces radicus*, cooling tower systems (KX090340)	99	MG754022
*Mortierella* sp.SA5.1	*Mortierella amoeboidea*, Indian Himalaya(MF467879)	99	MG754023
*Mortierella* sp.SA5.2	*Mortierella* sp., King George Island, Antarctic (JQ670951)	100	MG754024
*Mortierella* sp.SA5.3	*Mortierella* sp., Deception Island Antarctica (KC514910)	97	MG754025
*Peudogymnoascus* sp.SA5.5	*Peudogymnoascus* sp., Antarctic Peninsula (LC085196)	89	MG754026
*Rhizopus* sp.SA5.6	*Rhizopus microsporus*, Indonesian Tempeh Inoculant (KF709998)	99	MG845132
*Mortierella* sp.SA5.7	*Mortierella* sp., Ross Sea Antarctica region (DQ317354)	97	MG754027
*Rhizopus* sp.SA5.8	*Rhizopus microsporus*, maize rhizosphere soil (MF945552)	92	MG754028
*Ascomycota* sp.SA5.10	*Ascomycota* sp., Deception Island Antarctica (KC514882)	99	MG754029
*Mortierella* sp.SA6.1	*Mortierella* sp., Deception Island Antarctica (KC514910)	99	MG754030
*Rhizopus* sp.SA6.2	*Rhizopus microsporus*, maize rhizosphere soil (MF945552)	99	MG754031
*Antarctomyces* sp.SA6.3	*Antarctomyces pellizariae*, Antarctic Peninsula (KX576510)	99	MG845133
*Rhizopus* sp.SA6.4	*Rhizopus microsporus*, maize rhizosphere soil (MF945552)	76	MG754032
Uncultured fungus SA6.8	Uncultured fungus, Antarctic soil fungal (KU559753)	99	MG754033
*Mortierella* sp.SA7.1	*Mortierella* sp., Antarctic Peninsula (MG001404)	99	MG754034
*Peudogymnoascus* sp.SA7.3	*Pseudogymnoascus pannorum*, Arctic soil (MG000968)	99	MG754035
*Onygenales* sp.SA8.1	*Onygenales* sp., altitude lakes in the Indian trans-Himalayas (MF326613)	99	MG754036
*Rhizopus* sp.SA8.4	*Rhizopus microsporus*, maize rhizosphere soil (MF945552)	99	MG754060
*Mortierella* sp.SA8.5	*Mortierella* sp*., Humulus lupulus* (AY842393)	100	MG845134
*Mortierella* sp.SA8.7	*Mortierella* sp., Deception Island Antarctic (KC514910)	99	MG754037
*Cosmospora* sp.SA8.8	*Cosmospora* sp., King George Island, Antarctic (MG813394)	96	MG754038
*Cosmospora* sp.SA8.9	*Cosmospora viridescens*, King George Island, Antarctic (MG813394)	85	MG754039
*Rhizopus* sp.SA8.13	*Rhizopus microsporus*, maize rhizosphere soil (MF945552)	99	MG754055
*Pseudogymnoascus* sp.SA9.2	*Pseudogymnoascus* sp., King George Island, Antarctic (MG813409)	99	MG754042
*Mortierella* sp.SA9.3	*Mortierella* sp., forest soil of Poland (EF152521)	100	MG845135
*Geomyces* sp.SA9.4	*Geomyces* sp., Arctic soil (JN630629)	99	MG845138
*Pseudogymnoascus* sp.SA9.6	*Pseudogymnoascus* sp., King George Island, Antarctic (MG813409)	98	MG754043
*Rhizopus* sp.SA9.7	*Rhizopus microsporus*, maize rhizosphere soil, (MF945552)	100	MG754058
*Rhizopus* sp.SA9.8	*Rhizopus microsporus*, maize rhizosphere soil, (MF945552)	99	MG754044
*Rhizopus* sp.SA9.9	Biogas plant, Hydrolysis tank sludge (MF919345)	99	MG754045
*Tetracladium* sp.SA10.1	*Tetracladium* sp., Glacier National Park, British Columbia (KP411581)	94	MG754046
*Leptosphaeria* sp.SA10.2	*Leptosphaeria veronicae*, from *Veronica austriaca* plants (JF740255)	99	MG754047
*Lecanicidium* sp.SA10.3	*Lecanicillium attenuatum*, seawater gradients Antarctica Peninsula (KY786076)	98	MG754057
*Mortierella* sp.SA10.4	*Mortierella polygonia*, soil from Pit Clave Glacier National Park (KP411578)	100	MG845136
*Thelebolus* sp.SA10.7	*Thelebolus* sp., King George Island, Antarctic (MG813440)	99	MG754051
*Pseudogymnoascus* sp.SA11.1	*Pseudogymnoascus* sp., King George Island, Antarctic (MG813409)	100	MG754052
*Pseudogymnoascus* sp.SA11.2	*Pseudogymnoascus* sp., King George Island, Antarctic (MG813409)	99	MG754053
*Rhizopus* sp.SA11.3	*Rhizopus microsporus*, maize rhizosphere soil (MF945552)	85	MG754054
*Bjerkandera* sp.SA12.1	*Bjerkandera adusta*, National park of Chiloe (KF562019)	99	MG754059
*Geomyces* sp.SA12.2	*Geomyces* sp., Antarctic Peninsula (JN630629)	99	MG845137

*Blue rows indicate sequence isolated from cold environment*.

### Fungal Community Structures by PCR-DGGE

Total DNA was extracted from both fungal and soil samples using the PowerPlant® DNA isolation kit for plants and the PowerSoil® DNA Isolation Kit for soil (MO BIO Laboratories, Inc., CA) according to the manufacturer's instructions.

The fungal community composition was evaluated by denaturing gradient gel electrophoresis (PCR-DGGE) according to Iwamoto et al. (2000). First, touchdown PCR was performed with reagents supplied with GoTaq® Flexi DNA Polymerase (Promega, Co.) using the primer sets NS1 (5′-GTA GTC ATA TGC TTG TCT C-3′)/NS8 (5′-TCC GCA GGT TCA CCT ACG GA-3′). A second PCR with the primer sets NS7-GC (5′-GAG GCA ATA ACA GGT CTG TGA TGC-3, GC-clamp: CGC CCG GGG CGC GCC CCG GGC GGG GCG GGG GCA CGG GGG)/F1Ra (5′-CTT TTA CTT CCT CTA AAT GAC C-3′) was performed with 94°C for 1 min; followed by 30 cycles of 55°C for 1 min; and 72°C for 3 min; with a final extension at 72°C for 7 min. The primer set NS1/NS8 amplifies a 1700 bp fragment of the 18S rRNA gene and NS7-GC/F1Ra amplifies a 400 bp fragment nested within the NS1/ NS8 target. The DGGE analysis was performed using a DCode system (Bio-Rad Laboratories, Inc.). Twenty-five μL of PCR product was loaded onto a 6% (w/v) polyacrylamide gel with a 40–70% gradient (urea and formamide). The electrophoresis was run for 16 h at 75 V. The gel was then stained with SYBR Gold (Molecular Probes, Invitrogen Co.) for 30 min and photographed on a UV transilluminator. Clustering of DGGE banding profiles using a dendrogram was carried out using Phoretix 1D analysis software (TotalLab Ltd., UK). The *in silico* analysis was also used to estimate the bacterial diversity by richness (S) and the Shannon-Wiener index and dominance by the Simpson Index (D) represented by 1- D or 1- λ (Sagar and Sharma, 2012).

### Statistical Analyses

Data normality was analyzed according to Kolmogorov's test. Data were analyzed by a one-way analysis of variance (ANOVA) and compared by Tukey's test using SPSS software (SPSS, Inc.). Values are given as the means ± standard errors. Differences were considered significant when the *P*-value was lower than or equal to 0.01. For chemical soil parameters, all tests were performed in triplicate. For the fungal community composition, data normality was analyzed according to Kolmogorov's test. The similarity between bacterial communities was visualized with distance-based redundancy analyses (dbRDA) using Primer 7 software (Primer-E Ltd., Ivybridge, UK), which showed a Bray–Curtis similarity index at 60 and 30% and stress values <0.14 (Clarke, 1993). Values are given as the means ± standard errors. Differences were considered significant when the *P*-value was lower than or equal to 0.01.

## Results

### Chemical Parameters of Antarctic Soils

To determine the chemical composition of the 12 collected Antarctic soils, chemical analyses (C, N, and pH) were performed using triplicate samples of each soil (Table 1). In general, soil samples showed total C values from 0.10 (soil 10) to 2.65% (soils 1) and total N values from 0.01 (soils 4 and 10) to 0.36 (soil 1). Because of the low N content, soil 4 showed the highest C/N ratio (42.10) in comparison with the rest of the samples (from 5.53 to 15.80). The pH ranged from 5.46 (soils 7) to 7.23 (soil 4).

**Figure 2:**
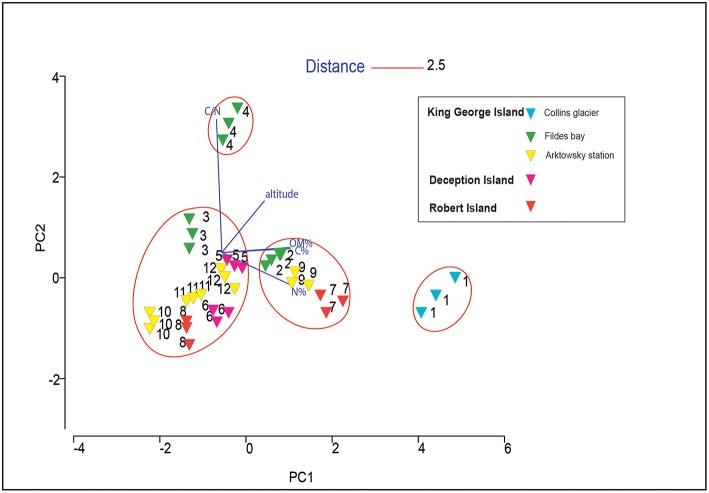
Principal component analysis (PCA) analysis based on the chemical properties of 12 Antarctic soil samples.

Principal component analysis showed that the soils were not grouped based on their location, where samples varied significantly (Figure 2). Soil 4 and soil 1 were grouped independently because of the high C/N ratio and high altitude of soil 4, and the high C and N content of soil 1. In soils 7 and 9, the C and N concentrations were also high, and along with their similar altitudes, they were grouped in the same cluster at a distance of 2.5. The other soil samples (soils 2, 3, 5, 6, 8, 10, 11, and 12) were similar to each other.

### Presence of Culturable Fungi

Our results revealed that in the pristine Antarctic environment, multiple culturable fungal taxa naturally occur. Thus, we isolated 54 fungal strains that, according to identification and phylogenetic affiliation based on the sequencing of the ribosomal internal transcribed spacer 2 (ITS2) region, belonged to 19 genera: *Penicillium, Pseudogymnoascus, Lambertella, Cadophora, Candida, Mortierella, Oxygenales, Geomyces, Vishniacozyma, Talaromyces, Rhizopus, Antarctomyces, Cosmospora, Tetracladium, Leptosphaeria, Lecanicillium, Thelebolus, Bjerkandera*, and an uncultured Zygomycete. Among these, 70% belonged to the Ascomycota, 10% to the Zygomycota, 10% to the Basidiomycota, 5% to the Deuteromycota and 5% to the Mucoromycota.

**Figure 3:**
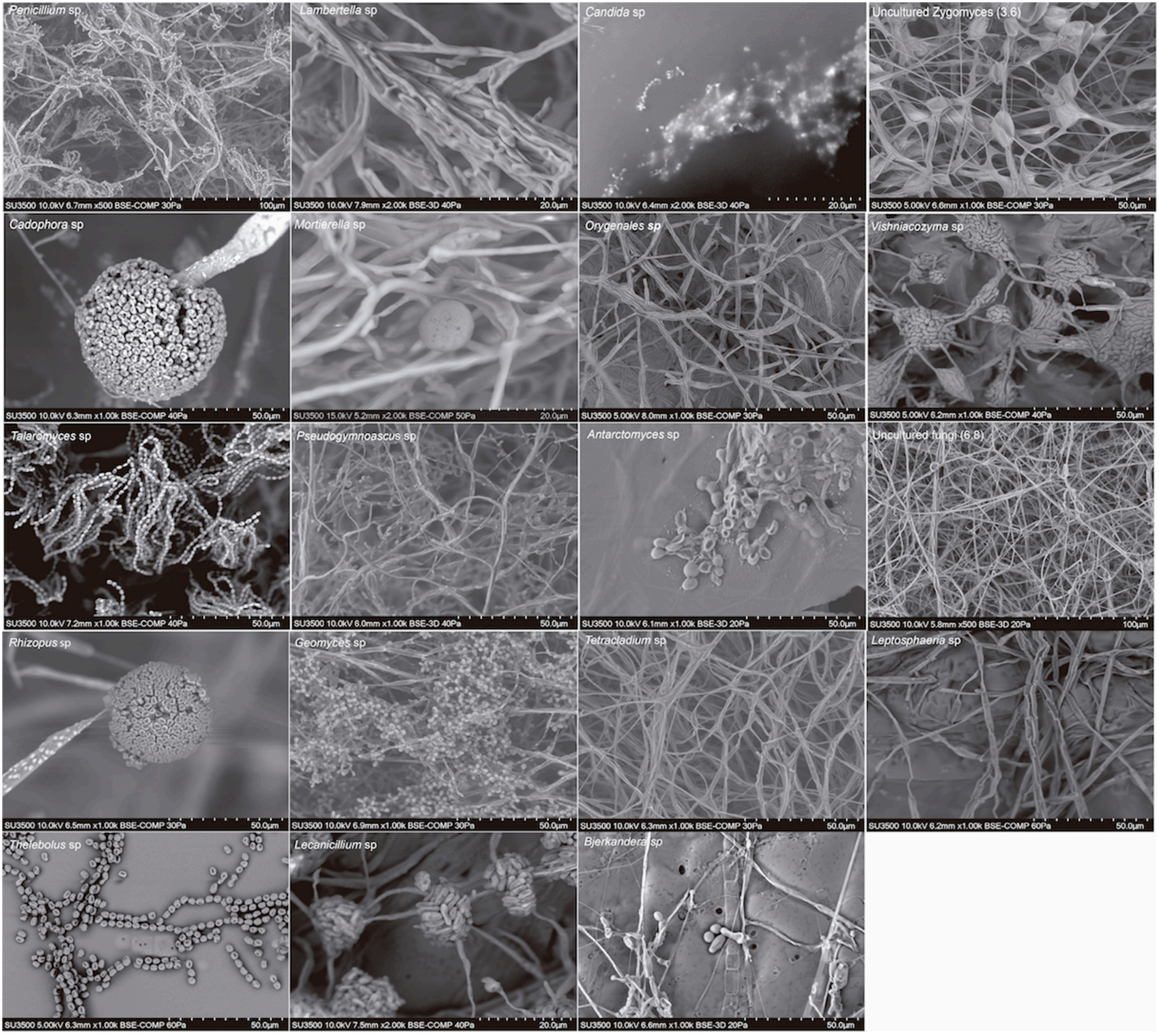
Scanning electron micrographs of genera of cultivable fungi isolated from South Shetland Islands.

We also found a wide variety of phenotypes, as was observed in scanning electron micrographs (SEM) of each genus (Figure 3). The SEM images from spores revealed the presence of teleomorph forms of the following genera from the Ascomycota: *Cadophora, Talaromyces, Antarctomyces, Thelebolus, Lecanicillium*; from the Basidiomycota: *Vishniacozyma* and *Bjerkandera* and from the Zygomycota: *Mortierella* and *Rhizopus*. The most frequently represented genus was *Mortierella*, and the soil samples with the highest richness of culturable fungi were soils 3, 5, 8, and 9 (Table 3).

**Table 3:** Presence of culturable fungal strains in each soil samples.

	**Soil Samples**												
**Strain**	**1**	**2**	**3**	**4**	**5**	**6**	**7**	**8**	**9**	**10**	**11**	**12**	**Total**
*Penicillium* sp.	1	1	–	–	–	–	–	–	–	–	–	–	2
*Pseudogymnoascus* sp.	1	–	–	–	1	–	1	–	2	–	2	–	7
*Lambertella* sp.	–	–	1	–	–	–	–	–	–	–	–	–	1
*Cadophora* sp.	–	–	3	–	–	–	–	–	–	–	–	–	3
*Candida* sp.	–	–	1	–	–	–	–	–	–	–	–	–	1
Uncultured Zygomycete	–	–	1	–	–	–	–	–	–	–	–	–	1
*Mortierella* sp.	–	–	1	1	4	1	1	2	1	1	–	–	12
*Onygenales* sp.	–	–	–	1	–	–	–	1	–	–	–	–	2
*Geomyces* sp.	–	–	–	1	–	–	–	–	1	–	–	1	3
*Vishniacozyma* sp.	–	–	–	1	–	–	–	–	–	–	–	–	1
*Talaromyce*s sp.	–	–	–	1	–	–	–	–	–	–	–	–	1
*Rhizopus* sp.	–	–	–	–	2	2	–	2	3	–	1	–	10
*Ascomycota* sp.	–	–	–	–	1	–	–	–	–	–	–	–	1
*Antarctomyces* sp.	–	–	–	–	–	1	–	–	–	–	–	–	1
Uncultured fungus	–	–	–	–	–	1	–	–	–	–	–	–	1
*Cosmospora* sp.	–	–	–	–	–	–	–	2	–	–	–	–	2
*Tetracladium* sp.	–	–	–	–	–	–	–	–	–	1	–	–	1
*Leptosphaeria* sp.	–	–	–	–	–	–	–	–	–	1	–	–	1
*Lecanicillium* sp.	–	–	–	–	–	–	–	–	–	1	–	–	1
*Thelebolus* sp.	–	–	–	–	–	–	–	–	–	1	–	–	1
*Bjerkandera* sp.	–	–	–	–	–	–	–	–	–	–	–	1	1
Total	2	1	7	5	8	5	2	7	7	5	3	2	54

Among culturable fungi, ~70% were associated with similar genera grown in cold environments according to GenBank database (Table 2, light blue rows). This was confirmed by the phylogenetic tree with representative 18S rRNA gene sequences (Supplementary Figure 1). Our isolates (blue letter) showed no similarity with other reported fungal strains belonging to the same genera growing in tropical or temperate areas (red letter).

### Optimal Fungal Growth Temperatures

Of the total culturable fungi, 55% were psychrotrophs and 45% were psychrophiles (Table 4). All strains were able to grow at 4°C, but psychrotrophs were able to grow above 20°C, whereas psychrophiles showed a maximum of growth at 15°C but were not able to grow at 25°C (Supplementary Figure 2).

**Table 4:** Fungal growth at different temperatures.

**Strain**	**Temperature of growth**			**Psychrophile/ psychrotroph**
	**4°C**	**15°C**	**25°C**	
*Penicilium* sp.	++	+++	++	Psychrotroph
*Pseudogymnoascus* sp.	+	+++	–	Psychrophile
*Lambertella* sp.	+	++	++	Psychrotroph
*Cadophora* sp.	+	+++	++	Psychrotroph
Unculture zygomycete	+	+	–	Psychrophile
*Mortierella* sp.	+	++	++	Psychrotroph
*Onygenales* sp.	+	++	–	Psychrophile
*Geomyces* sp.	+	++	–	Psychrophile
*Vishniacozyma* sp.	+	+++	–	Psychrophile
*Talaromyces* sp.	+	+++	–	Psychrophile
*Rhizopus* sp.	+	+	–	Psychrophile
*Ascomycota* sp.	+	+	–	Psychrophile
*Antarctomyces* sp.	+++	+	+	Psychrotroph
Uncultures fungus	+	++	+	Psychrotroph
*Cosmospor*a sp.	+	+++	++	Psychrotroph
*Tetracladium* sp.	+	+++	++	Psychrotroph
*Leptosphaeria* sp.	+	+++	–	Psychrophile
*Lecanicidium* sp.	+	+++	++	Psychrotroph
*Thelebolus* sp.	+	++	++	Psychrotroph
*Bejerkendera* sp.	+	+++	+++	Psychrotroph

*Growth rate capacity was measured as follows: +++, very high capacity; ++, high capacity; +, normal capacity; and –, no capacity*.

### Fungal Community Composition in Antarctic Pristine Environment

**Figure 4:**
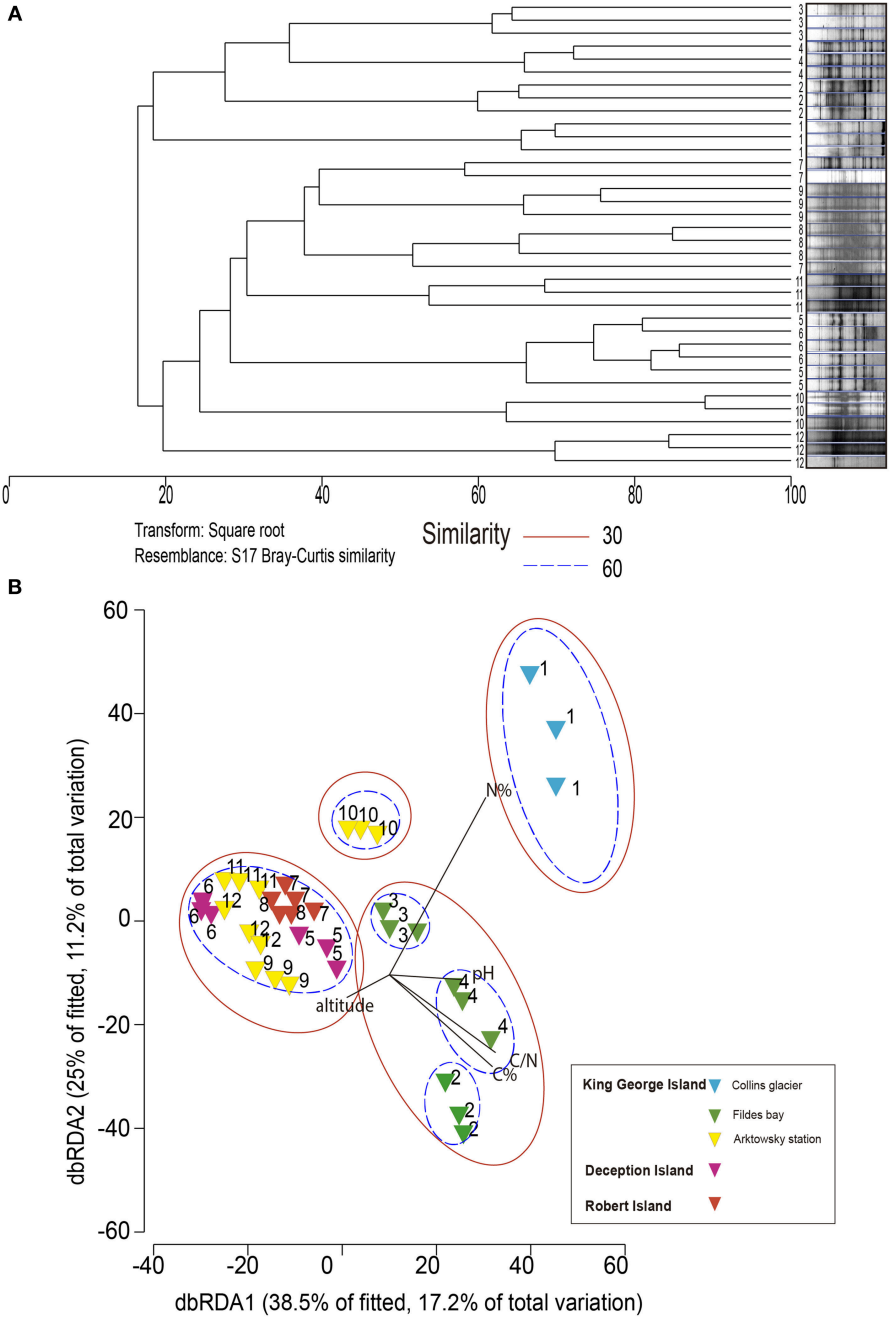
Dendrogram **(A)** and nonmetric multidimensional scaling **(B)** analysis of DGGE profiles (18S rRNA gene) from soil communities of the Antarctic pristine environment.

The dominance and diversity of fungal community composition was not related to soil location as revealed by dbRDA, with which microbiological and soil chemical/environmental properties were analyzed (Figure 4). The fungal community structure of sample 1 (Collins Glacier) was grouped independently and was influenced by high N content at 60% similarity. The fungal community of sample 10 was also grouped independently, probably due to the lower altitude and N content compared with the rest of the samples. At 60% similarity, the communities of soils 2, 3, and 4 were grouped independently, but at 30% similarity, they were grouped with the rest of the samples (soils 5, 6, 7, 8, 9, 11, and 12) without significant differences among their communities.

### Fungal Richness and Diversity and Relation to Chemical Parameters

**Figure 5:**
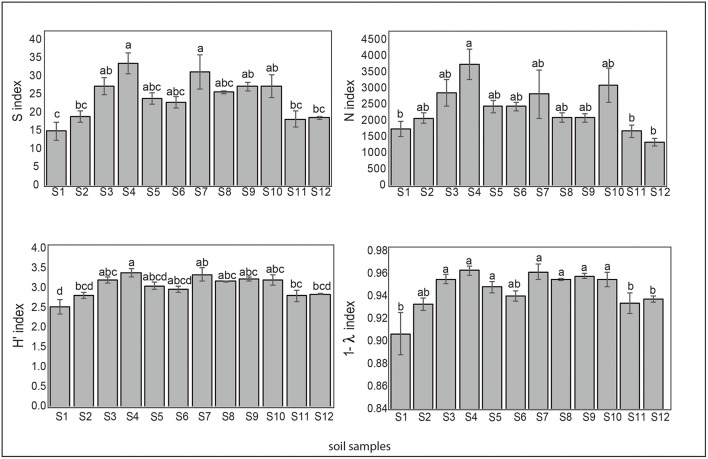
Biodiversity indices S (species number), N (individual number), Shannon-Wiener (H) and Simpson (represented by 1- λ) of the soil samples. Tukey's test was used to compare treatment means, and values followed by the same letter do not differ at *P* < 0.05 (*n* = 5).

Regarding the microbial diversity in the pristine Antarctic environment, in general, samples 1 (Collins Glacier), 2 (Fildes Bay), 11 and 12 (Arctowski Station) showed less richness expressed in species number (S), which ranged from 15 to 33. A similar tendency was observed for N (individual number), ranging from 1,500 to 3,500. The most richness was observed in samples 4 (Fildes Bay), 7 (Coppermine Peninsula) and 10 (Arctowski Station). The Shannon index (H′) that represents richness and dominance ranged from 2.5 to 3.3. Likewise, samples 1, 2, 11, and 12 showed major dominance expressed as Simpsons (D) index represented by 1-ƛ (Figure 5).

**Figure 6:**
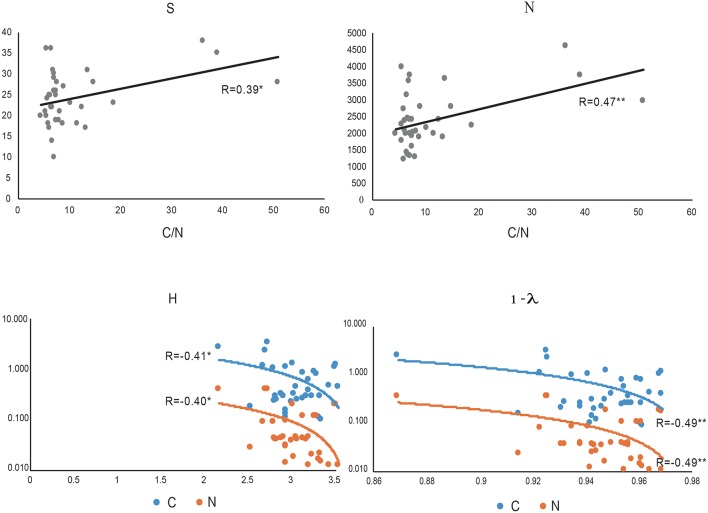
The most important correlation between biodiversity index: S (species number), N (individual number, Shannon-Wiener (H) and Simpson (represented by 1- λ) and C/N ratio (gray), C (blue) and N (orange).

We noted that a positive correlation exists between C/N and index of richness expressed as S (species number) and N (individual number) (*p* < 0.05^*^ and *p* < 0.01^**^, respectively, Figure 6). The index related to richness and dominance such as the Shannon Wiener index (H') and the index related to dominance only, such as the Simpsons (D) index, represented by 1-ƛ was inversely related to C and N. Thus, we noted high dominance (low diversity) when soil samples showed higher C and N contents (Figure 6).

## Discussion

During the Antarctic campaign ECA-53, 12 bulk soils (0–20 cm) were collected from South Shetland Island (62°00′S, 59°30′W), eight from King George Island (including Collins Glacier), two from Deception Island and two from Robert Island. According to the chemical parameter measurements, pH values were near neutral, from 5.46 to 7.23, with C ranging from 0.10 to 2.65% and N ranging from 0.01 to 0.36%, and Collins Glacier showed the greatest values. Moorhead et al. (2003) showed that wetlands in Taylor Valley (Antarctica) showed an accumulation of soil organic C and total N concentrations of 0.12% and 0.013%, respectively. However, pH values were more alkaline than in our study (~8.5). Bölter et al. (1997) reported values of pH more acidic, from 3.7 to 7.2 for Arctowski Station and values of 0.09 to 2.60% C and 0.16 to 0.48% N. This variation could be attributed to the high influence of sea birds (penguins) and other birds (Lee et al., 2009).

Fifty-four fungal strains belonging to 20 genera were isolated from King George Island (including Collins Glacier), Deception and Robert Island soils. The predominance of filamentous Ascomycetes (70%) in studies of Antarctic soils has been reported, with *Geomyces* and *Cadophora* widely reported (Högberg et al., 2007). We also found the presence of *Zygomycota, Basidiomycota, Deuteromycota*, and *Mucoromycota*. Similar genera isolated from Antarctic and Arctic environments were reported in other studies by Krishnan et al. (2018), and according to our study, 70% of isolates were associated with similar genera grown in cold environments according to the GenBank database (Supplementary Figure 1). Ding et al. (2016) reported high dominance of *Pseudogymnoascus* (or *Geomyces*) in Fildes Peninsula and *Antarctomyces* and *Thelebolus*, which are considered cold-environment-specific genera (Ding et al., 2016). Thus, among all culturable isolates from our study, 100% were able to grow at 4°C, 55% were considered psychrotrophic fungi that were able to grow above 20°C, whereas psychrophilic microorganisms (45%) showed maximum growth at 15°C but were not able to grow at 25°C (Robinson, 2001).

Interestingly, we noted less richness and major dominance in samples collected in Collins Glacier compared with the rest of the soils analyzed, despite the large content of C and N. However, the C/N ratio seems to be a more important parameter influencing soil fungal richness because our results showed that a positive correlation exists between C/N and the index of richness expressed as S (species number) and N (individual number). Calandra et al. (2016) showed that the C:N ratio had an important role in spore shelf life of *Trichoderma harzianun*. Similarly, Dumbrell et al. (2010) showed that ß-diversity of arbuscular mycorrhizal fungi was positively correlated with the C/N ratio. However, the indices related to richness and dominance, such as the Shannon Wiener index (H′) and dominance only, such as Simpsons (D) were inversely related to C and N. Therefore, high fungal dominance (low diversity) was found when soil samples showed a larger C and N content. In this context, Siciliano et al. (2014) reported that soil fertility (i.e., organic matter, nitrogen and chloride content) was consistently the most important driver influencing bacterial and fungal species richness, speculating that soil fertility provides nutritive properties that allow the more adapted species within a community to grow rapidly and to dominate and exclude other members of the community. Thus, it has been reported that the Antarctic microbial community seems to be structured solely by abiotic processes (Cary et al., 2010). Altitude was not a determinate parameter influencing the fungal community, as was the case in a study recently reported by Coleine et al. (2018).

In relation to soil fungal community compositions, our results showed that soil location was not a parameter that defined the microbial community structure, as revealed by dbRDA, as soil parameters were the most prominent influencing factor. For example, soil collected from Collins Glacier (sample 1) was strongly influenced by N and sample 10 by low N and low altitude. Siciliano et al. (2014) noted that soil pH was a major factor determining the bacterial community composition in polar soil because fungi can adapt to different pH ranges. In the case of fungal community compositions, we found that C content was the most important factor.

Because PCR-DGGE is a simple and relatively inexpensive method, it is considered a valuable tool to detect gross shifts in the entire microbial community (Hume et al., 2011; Rychlik et al., 2016). However, further studies considering omics technology (such as genomics, transcriptomics, proteomics, etc.) are needed to identify functional roles of fungal adaptations to extreme environments in order to better understand the endemic nature of these communities for further biotechnological applications.

## Conclusions

Our results reveal fungi occurrence in twelve soils from South Shetland Island, Antarctica. The most representative group were the Ascomycetes (70%), with *Geomyces* and *Cadophora* widely reported in Antarctic lands. Seventy percentage of isolates were associated with similar genera grown in cold environments, and among the total culturable fungal strains, 55% were considered psychrotrophic (able to grow above 20°C), and 45% were considered psychrophilic with a maximum growth at 15°C and no growth at 25°C. Remarkably, the occurrence of fungi in soils was not correlated with soil locations but was correlated with soil chemical properties. Thus, richness was associated with the C/N ratio, and dominance was inversely correlated with C and N. Most fungal strains were observed in the teleomorph phase, with clear survival structures to allow resistance to extreme environments. Future omic analyses are required to identify the ecological role and fungal adaptation mechanisms at low temperature as well as the to examine potential applications of these fungal strains in biotechnology.

## Author Contributions

PD wrote the main manuscript text. MJ, SV, and PB designed the research. CP, MM, MJ, and RB supervised the study and improve the revision, and RB, CF, CP, and PD analyzed the data. All authors critically revised the manuscript and approved the final version.

### Conflict of Interest Statement

The authors declare that the research was conducted in the absence of any commercial or financial relationships that could be construed as a potential conflict of interest.
