# Supplementary material for: Occurrence of Soil Fungi in Antarctic Pristine Environments
Source: Front Bioeng Biotechnol. 2019 Mar 7;7:28. doi: 10.3389/fbioe.2019.00028 (PMC6416174; doi:10.3389/fbioe.2019.00028)
Supplement: Supplementary file 1 [file Data_Sheet_1.PDF]

Supplementary Figures

Supplementary Figure 1. Phylogenetic tree showing the affiliation of cultivable fungi isolated in this study from the South Shetland Islands (blue letter). The neighbor-joining tree was constructed with representative 18S rRNA gene sequences of the same genera isolated from tropical areas (red letter). A bootstrap analysis was performed with 1,000 runs. The accession numbers are in parentheses.

Supplementary Figure 2. Psychrophile (green line) and psychrotroph (yellow line) fungal growth at 4ºC, 15ºC and 25ºC.

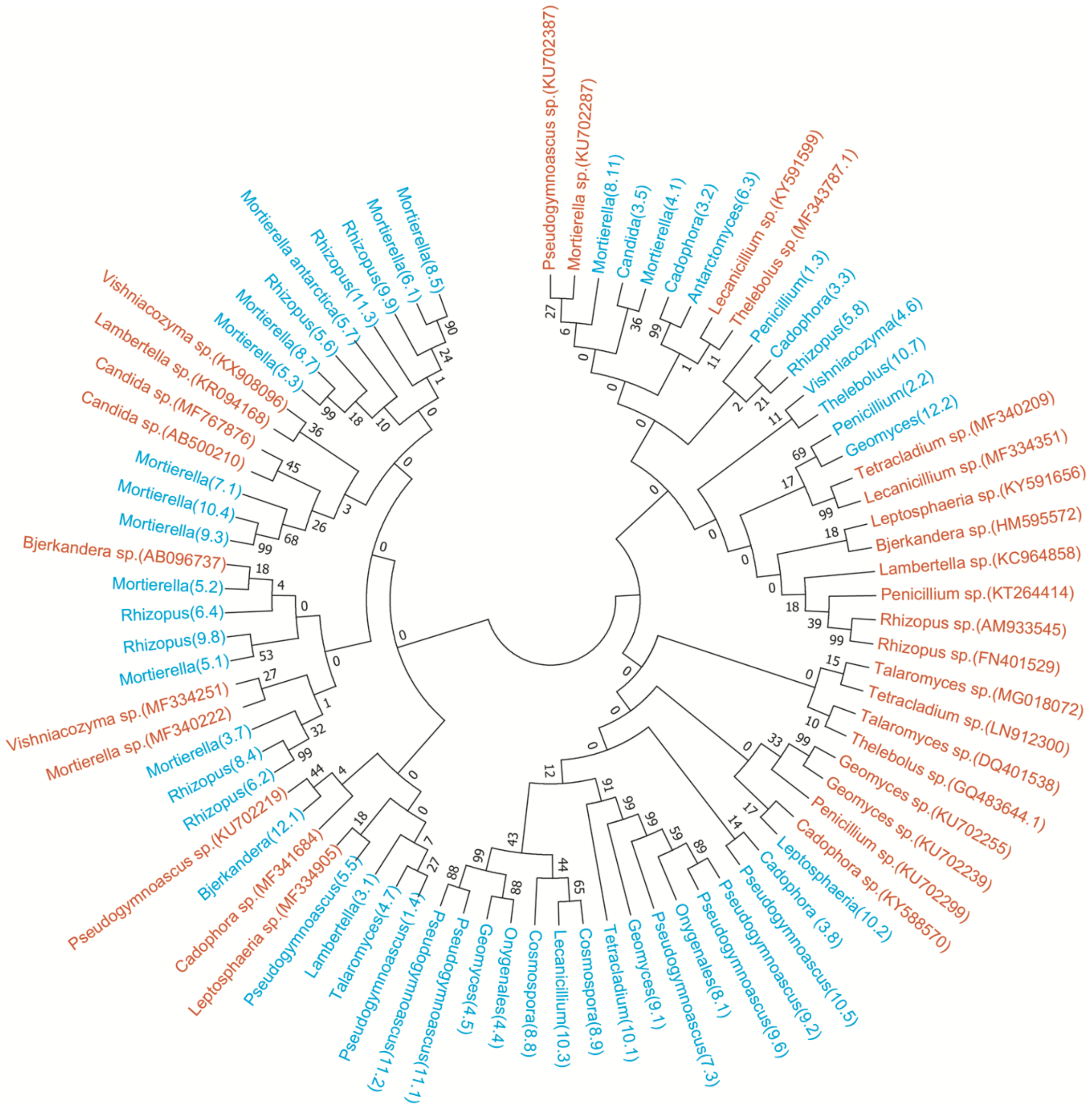
Pseudogymnoascus sp.(KU702387)
Mortierella sp.(KU702287)
Mortierella(8.11)
Candida(3.5)
Mortierella(4.1)
Cadophora(3.2)
Antarctomyces(6.3)
Lecanicillium sp.(KY591599)
Thelebolus sp.(MF343787.1)
Penicillium(1.3)
Cadophora(3.3)
Rhizopus(5.8)
Vishniacozyma(4.6)
Thelebolus(10.7)
Penicillium(2.2)
Geomyces(12.2)
Tetracladium sp.(MF340209)
Lecanicillium sp.(MF334351)
Leptosphaeria sp.(KY591656)
Bjerkandera sp.(HM595572)
Lambertella sp.(KC964858)
Penicillium sp.(KT264414)
Rhizopus sp.(AM933545)
Rhizopus sp.(FN401529)
Talaromyces sp.(MG018072)
Tetracladium sp.(LN912300)
Talaromyces sp.(DQ401538)
Thelebolus sp.(GQ483644.1)
Geomyces sp.(KU702255)
Geomyces sp.(KU702239)
Penicillium sp.(KU702299)
Cadophora sp.(KY588570)
Leptosphaeria(10.2)
Cadophora (3.8)
Pseudogymnoascus(10.5)
Pseudogymnoascus(9.2)
Pseudogymnoascus(9.6)
Onygenales(8.1)
Pseudogymnoascus(7.3)
Geomyces(9.1)
Tetracladium(10.1)
Cosmospora(8.9)
Lecanicillium(10.3)
Cosmospora(8.8)
Onygenales(4.4)
Geomyces(4.5)
Pseudogymnoascus(11.1)
Pseudogymnoascus(11.2)
Pseudogymnoascus(1.4)
Talaromyces(4.7)
Lambertella(3.1)
Pseudogymnoascus(5.5)
Leptosphaeria sp.(MF334905)
Cadophora sp.(MF341684)
Bjerkandera(12.1)
Pseudogymnoascus sp.(KU702219)
Rhizopus(6.2)
Rhizopus(8.4)
Mortierella(3.7)
Mortierella sp.(MF340222)
Vishniacozyma sp.(MF334251)
Mortierella(5.1)
Rhizopus(9.8)
Rhizopus(6.4)
Mortierella(5.2)
Bjerkandera sp.(AB096737)
Mortierella(9.3)
Mortierella(10.4)
Mortierella(7.1)
Candida sp.(AB500210)
Candida sp.(MF767876)
Lambertella sp.(KR094168)
Vishniacozyma sp.(KX908096)
Mortierella(5.3)
Mortierella(8.7)
Rhizopus(5.6)
Mortierella antarctica(5.7)
Rhizopus(11.3)
Rhizopus(9.9)
Mortierella(6.1)
Mortierella(8.5)

Supp. Fig. 1

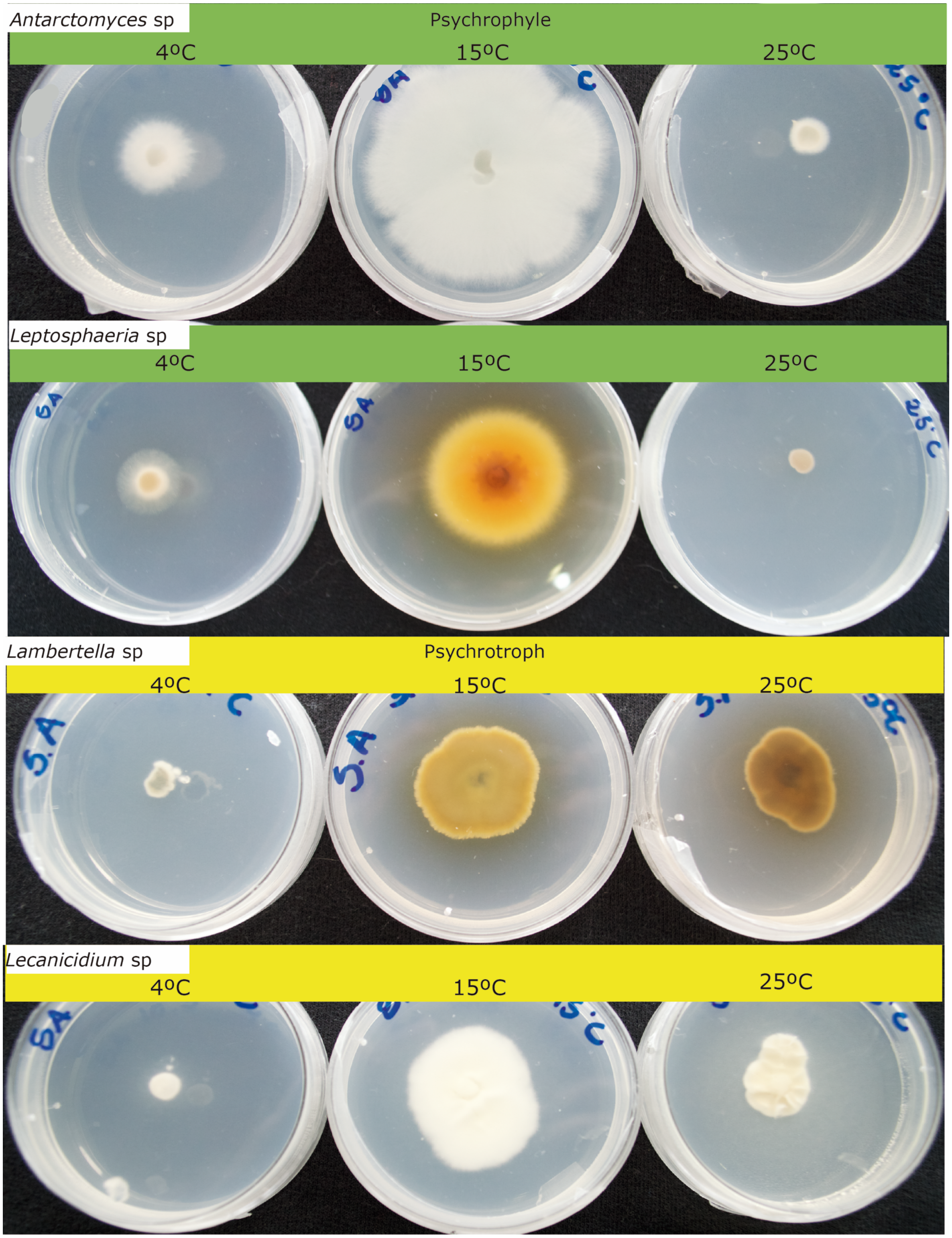
Antarctomyces sp
Psychrophyle
4ºC
15ºC
25ºC
Leptosphaeria sp
4ºC
15ºC
25ºC
Lambertella sp
Psychrotroph
4ºC
15ºC
25ºC
Lecanicidium sp
4ºC
15ºC
25ºC

Supp. Fig. 2
